# Design of a Quencher-Free Fluorescent Aptasensor for Ochratoxin A Detection in Red Wine Based on the Guanine-Quenching Ability

**DOI:** 10.3390/bios12050297

**Published:** 2022-05-05

**Authors:** Cheng Yang, Fathimath Abbas, Amina Rhouati, Yingying Sun, Xiaolin Chu, Shengnan Cui, Bingbing Sun, Changying Xue

**Affiliations:** 1 State Key Laboratory of Fine Chemicals, Department of Chemistry, School of Chemical Engineering, Dalian University of Technology, Dalian 116024, China; yangcheng@dlut.edu.cn (C.Y.); ferthunabbas@mail.dlut.edu.cn (F.A.); sunyydlut@hotmail.com (Y.S.); 21907036@mail.dlut.edu.cn (X.C.); cuishengnan@mail.dlut.edu.cn (S.C.); bingbingsun@dlut.edu.cn (B.S.); 2 Bioengineering Laboratory, Higher National School of Biotechnology, Constantine 25100, Algeria; amina.rhouati@gmail.com; 3 State Key Laboratory of Fine Chemicals, School of Bioengineering, Dalian University of Technology, Dalian 116024, China

**Keywords:** aptasensor, quencher-free, photo-induced electron transfer, guanine-quenching fluorescence, ochratoxin A

## Abstract

This study describes a quencher-free fluorescent aptasensor for ochratoxin A (OTA) detection using the specific quenching ability of guanine for fluorescein (FAM) molecules based on photo-induced electron transfer (PIET). In this strategy, OTA is detected by monitoring the fluorescence change induced by the conformational change of the aptamer after target binding. A new shorter OTA aptamer compromising three guanine bases at the 5′ end was used in this study. This new aptamer, named G3-OTAapt1-FAM (F1), was labeled with FAM on the 3′ end as a fluorophore. In order to increase the binding affinity of the aptamer and OTA, G3-OTAapt2-FAM (F2) was designed; this added a pair of complementary bases at the end compared with F1. To prevent the strong self-quenching of F2, a complementary chain, A13, was added. Although the F1 aptasensor was simpler to implement, the sensitivity of the F2 aptasensor with A13 was better than that of F1. The proposed F1 and F2 sensors can detect OTA with a concentration as low as 0.69 nmol/L and 0.36 nmol/L, respectively.

## 1. Introduction

Aptamers are folded nucleic acid sequences with a single-stranded DNA or RNA structure, designed from 25 to 60 base pairs. They are selected via an in vitro process called the systematic evolution of ligands by exponential enrichment (SELEX) from an extensive random library with desirable properties. The sequence variation allows for the display of many structural arrangements; therefore, aptamers can form secondary structures that specifically bind to some targets, such as small molecules, proteins, amino acids, cells, and tissues, with a high affinity [1]. Aptamers are characterized by a target-induced conformational change that folds into stable three-dimensional structures, such as a hairpin, pseudo-knot, and G-quadruplex [2]. Aptamers have been identified as a promising alternative to replace antibodies because of their similar recognition function [3]. In addition, aptamers are superior because they offer several advantages over antibodies, which include easy modification, high affinity, and stable thermal and chemical stability. In addition, aptamers are selected by in vitro techniques, independent of animals or cells, which contribute to their low-cost production [4,5]. These make them a promising molecular receptor in bioanalytical applications [3]. Based on the outstanding merits of aptamers, many aptasensors have been developed for different applications, such as electrochemistry [6,7], fluorescence [7,8], chemiluminescence [6,9], and colorimetry [6,7,10] assay approaches. Fluorescence-based aptasensors have attracted plenty of attention because they possess various advantages, including a convenient operation, quick response, and good reproducibility [11,12]. Nevertheless, the challenge for fluorescence methods is the high cost of labeling aptamers with the fluorophore and quencher.

Guanine is an excellent quenching group for quencher-free aptasensors. Oligonucleotides rich in guanines have been employed as sensing elements in various biosensors over the past few years [13]. Guanine is the most oxidizable base; this base–dye interaction is believed to be caused by the photo-induced electron transfer (PIET) mechanism [14,15]. Thus, the fluorescence of the fluorophore can be quenched by the neighboring guanine [13,16]. Recently, DNA, RNA, and metal ions analyses have emerged, based on PIET, between fluorophore and guanine bases [13,16,17]. For these methods, guanine is used as a quencher for FAM, which avoids employing quencher unit binding to the oligonucleotide, such as hemin or porphyrin. Conjugating an organic fluorescence quenching group such as BHQ1 (black hole quencher 1) or TAMRA for fluorescence detection increases the complexity of the detection method, and significantly increases the cost of testing [18]. Guanine is a low-cost and effective quencher for FAM. Moreover, guanines can be easily added to the aptamer sequence without the complex conjugations required for organic quenchers. Therefore, we tried to use this strategy to detect highly toxic small molecules, which are challenging to detect with a high sensitivity.

Ochratoxin A (OTA) is a mycotoxin that is considered a human carcinogen by the International Agency for Research on Cancer (IARC) [4]. OTA is found in various foodstuffs, including grains, coffee, wine, and beer. Certain fungal species produce ochratoxins as a secondary metabolite, such as *Aspergillus ochraceus*, *Aspergillus carbonarius,* and *Penicillium verrucosum.* Because of the extensive existence and chemically stable properties of OTA (even surviving at high temperatures and boiling), it is tough to eradicate from the food chain. Hence, the accumulation of OTA in the human body causes a potential threat to human health. Therefore, specific regulatory bodies have set strict food and feed regulations regarding the OTA content. The European Commission has stipulated that the maximum content of OTA is 2 µg/kg in grape juice or wine and 5 µg/kg and 10 µg/kg in raw grains and soluble coffee, respectively [19,20]. Therefore, it is essential to develop a new approach to detect OTA in a highly efficiency and low-cost manner. The application of aptamers has brought hope to this expectation. In 2008, Cruz-Aguado selected the first OTA aptamer for OTA detection [21]; since then, this aptamer has been widely used to construct many biosensors [11,22,23,24]. Among these, fluorescence aptasensors are the simplest, easiest to operate, and are extremely sensitive. Therefore, we have taken advantage of the excellent properties of aptamers, together with the quenching ability of guanine, to develop a fluorescence aptasensor for OTA detection using guanine as a quencher. For this purpose, a new aptamer with a blunt end was used on the platform. FAM as a fluorophore was labeled on the 3′ end of the new aptamer, and three guanine bases as a quencher were extended at the 5′ end (named G3-OTAapt1-FAM (F1)). In order to increase the binding affinity of the aptamer and OTA, a new aptamer called G3-OTAapt2-FAM (F2) was designed, which added a pair of complementary bases at the end of F1. When the target molecule OTA is present, the aptamer forms a recognition structure. It places FAM closer to the protruding guanines, leading it to be quenched by the guanines. As a result, OTA can be detected quantitatively through fluorescence quenching.

In this study, based on the quenching ability of guanine, we developed a fluorescence analysis method for OTA detection that is highly sensitive, and has a high throughput and low cost.

## 2. Experimental

### 2.1. Reagents and Chemicals

Sodium chloride (NaCl), calcium chloride (CaCl_2_), and hydrochloric acid (HCl) were purchased from Sinopharm Chemical Reagent Co., Ltd. (Beijing, China). Tris (hydroxymethyl) carbamate (Tris, C_4_H_11_NO_3_) was purchased from BBI Life Sciences (Shanghai, China). OTA, OchratoxinB (OTB), Aflatoxin B1 (AFB1), Fumonisin B1 (FB1), Deoxynivalenol (DON), and Zearalenone (ZEA) were purchased from Sigma-Aldrich (St. Louis, MO, USA). All of the oligonucleotides (listed in Table 1) were synthesized by Shanghai Sangon Biotechnology Co., Ltd. (Shanghai, China), and were annealed by heating at 95 °C for 5 min, and then quickly cooled down to 4 °C and maintaining this for 2.5 min before use. The ultra-pure water used in the experiment was purified by a Milli-Q system (18.2 MΩ; Millipore, Bedford, MA, USA). The 96-well micro-plates were purchased from Shanghai Wohong Biotechnology Co., Ltd. (Shanghai, China).

### 2.2. Instrumentation

All of the fluorescence measurements were performed at room temperature on a Safire II multi-detection micro-plate reader (Tecan, Switzerland), with an excitation wavelength of 483 nm and an emission wavelength of 525 nm. The concentration of the aptamer was determined using UV absorption spectroscopy by a ReadMax 1900 Absorbance Microplate Reader (Shanghai Flash Spectrum Biotechnology Co., Ltd., Shanghai, China).

### 2.3. Experimental Method

#### 2.3.1. Optimization of Reaction Conditions

In order to achieve the best experimental performances, the concentration of Ca^2+^, the complementary sequence, and the concentration of the complementary chain were optimized. The optimal concentration of Ca^2+^ was determined by mixing different concentrations of Ca^2+^ (0–100 nmol/L) with 20 nmol/L F1, 10 mmol/L Tris-HCl (pH = 8.4), and 120 mmol/L NaCl in the presence and absence of 50 nmol/L OTA at room temperature. To obtain the best complementary sequence, the calibration curves were obtained using different complementary sequences (A11–A18) of 60 nmol/L and 20 nmol/L F2 in 10 mmol/L Tris-HCl (pH = 8.4), 120 mmol/L NaCl, and 3 mmol/L CaCl_2_ at room temperature. To determine the optimal concentration of the complementary sequence, different concentrations of A13 (0–1000 nmol/L) and 20 nmol/L F2 with 10 mmol/L Tris-HCl (pH = 8.4), 120 mmol/L NaCl, and 3 mmol/L CaCl_2_ were mixed in the presence and absence of 50 nmol/L of OTA at room temperature. The fluorescence intensity was obtained using a Safire II multi-detection microplate reader, and all of the experiments were repeated three times independently.

#### 2.3.2. OTA Aptasensing by Fluorescence Aptasensor

The testing solution for the F1 aptasenor was prepared by mixing 100 nmol/L F1 in a binding buffer (50 mmol/L Tris-HCl buffer solution containing 600 mmol/L NaCl and 15.0 mmol/L CaCl_2_, pH = 8.4), and the F2 aptasensor was prepared by mixing 100 nmol/L F2 and 300 nmol/L A13 complementary chains in the binding buffer. Then, 80 μL of OTA with different concentrations was added into 96-well micro-plates. Then, 20 μL of the testing solution was added to the micro-plates and mixed well to a final volume of 100 μL. Finally, the fluorescence intensity was measured using a Safire II multi-detection microplate reader after 20 min of incubation. All of the experiments were repeated three times independently.

#### 2.3.3. Specificity Assay

To verify the specificity of the developed aptasensor, OTB, AFB1, DON, FB1, and ZEA were used as the control samples. Then, 80 μL of the 62.5 nmol/L OTA solution or 250 nmol/L of the OTB, AFB1, DON, FB1, and ZEA solution was mixed with a 20 μL testing solution (described in Section 2.3.2) and incubated for 20 min at room temperature. The fluorescence intensity was determined using a Safire II multi-detection microplate reader, and all of the experiments were repeated three times independently.

#### 2.3.4. Detection of OTA in Wine Samples

In a standard addition experiment, 1.0 μL, 2.0 μL, and 4.0 μL of 10 μmol/L OTA standard solutions were added to 10 mL of red wine samples, seperately. The spike wine samples were obtained with 1.0 nmol/L, 2.0 nmol/L, and 4.0 nmol/L of OTA. The samples were pre-treated using the following process. According to the physical and chemical properties of OTA, sample pre-treatment was performed using a double liquid−liquid extraction scheme [25]. The operation process was as follows: 5.0 mL of red wine samples were mixed with an equal volume of toluene. After complete phase separation, 4.0 mL of the top organic layer was mixed with equal volumes of the alkaline buffer solution (10 mmol/L Tris-HCl, pH = 8.4). Then, complete phase separation was allowed. Then, 3.0 mL of the bottom aqueous layer was taken out and mixed with an equal volume of dichloromethane. Before mixing, the pH of the bottom aqueous layer was adjusted to 3.0 by adding hydrochloric acid. The two phases were allowed complete phase separation, and the bottom organic layer was collected and mixed with an equal volume of 10 mmol/L Tris-HCl buffer (pH = 8.4). Finally, after complete phase separation, the top aqueous phase was taken and used for subsequent analysis by the aptasensor.

Finally, 80 μL of the pre-treated samples were mixed and incubated with 20 μL of the testing solution (described in Section 2.3.2). The fluorescence intensity was detected by a Safire II multi-detection microplate reader, and all of the experiments were repeated three times independently.

## 3. Results and Discussion

### 3.1. Principle of OTA Detection

**Scheme 1:**
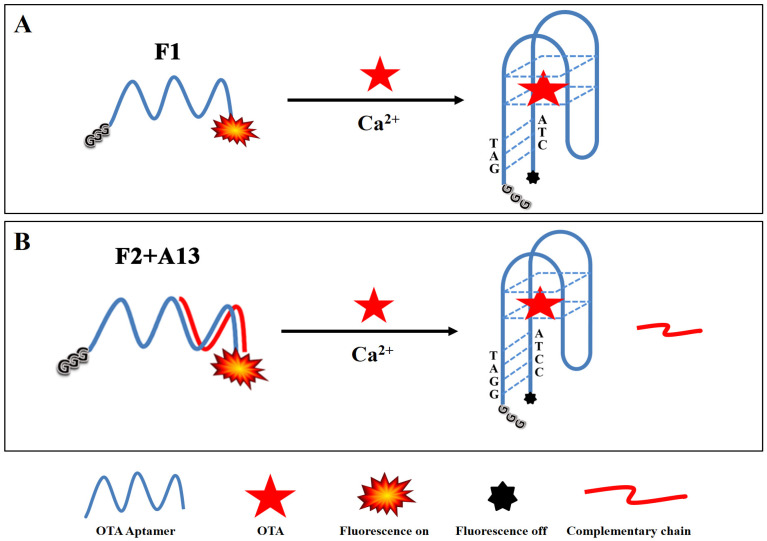
Schematic illustration of the guanine-quenched fluorescence sensing platform F1 biosensor (**A**) and F2 + A13 biosensor (**B**) for the detection of OTA.

The principle of the proposed fluorescence assay for the detection of OTA is depicted in Scheme 1. First, the aptasensor was formed using the aptamer selected by Cruz-Aguado, the FAM was attached at the 3′ end, and three guanines were extended at the 5′ end as a quencher. Unfortunately, no fluorescence quenching was observed, even after increasing the OTA concentration. The reason for this is likely that the aptamer selected by Cruz-Aguado has five bases at the 3′ end that are not required for OTA binding, which increases the distance between the FAM and the quencher. This is consistent with the previous research results of our group [12]. Therefore, these bases were truncated from the 3′ end of the original aptamer (OTAapt36) to create a new blunt end aptamer. The new aptamer was labelled with FAM on the 3′ end and extended using three guanine bases at the 5′ end, which was named G3-OTAapt1-FAM (F1).

For the new aptamer, F1, the presence of OTA caused the aptamer to form a compact three-dimensional structure (an antiparallel G-quadruplex), making the bases of the two tails of the aptamer close to each other, forming a hybridization structure [12]. As a result, FAM fluorescence could be quenched by three unpaired guanines protruding at the 5′ end. The distance between the FAM molecule and guanine bases decreased during this process, leading to fluorescence quenching by photo-induced electron transfer (PIET).

In order to further increase the binding affinity of the aptamer with OTA and lower the detection limit, a second aptamer was designed based on the fact that extending the number of complementary bases could stabilize a double-strand sequence. This aptamer was designed by adding a pair of G-C complementary bases at the end of the shorter OTA aptamer (31 bases), which was named G3-OTAapt2-FAM (F2). The F2 sequence has one more complementary base than F1, making the structure more stable and providing a higher affinity and quenching efficiency.

**Figure 1:**
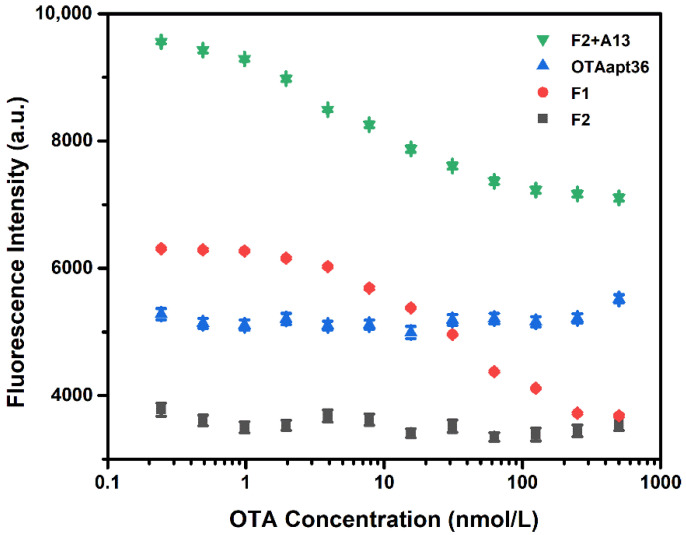
Comparison of the fluorescence intensity of the F1-based aptasensor and F2-based aptasensor with different OTA concentrations (*n* = 3). The concentration of the F1, F2, and OTAapt36 aptamers was 20 nmol/L, and the concentration of A13 was 60 nmol/L. The error bars denote the standard deviation of the triplicate measurements.

To demonstrate the feasibility of our aptasensor, the fluorescence property of the sensing system was investigated. As shown in Figure 1, no significant fluorescence quenching change was noted after adding OTA when using the original aptamer. This further validated our previous research results [12], that five bases at the 3′ end widened the distance between FAM and the quencher, which is not helpful for OTA detection. Thus, five bases had to be truncated from the 3′ end of the original aptamer (OTAapt36) to create a blunt end aptamer. As expected, after using the blunt tail aptamer (F1), better results were obtained. Although F2 was designed for further improvement, no significant fluorescence intensity change was noted when OTA was present. This was because of the tail chains of the newly designed aptamer already being hybridized with each other, even in the absence of OTA. Therefore, complementary chains were introduced into this detection system to prevent this occurrence. In general, the competition of complementary chains reduces the performance of the aptasensor, but here, the complementary chain amplifies the response without reducing the performance of the F2 aptasensor.

### 3.2. Optimization of OTA Detection Conditions

#### 3.2.1. Optimization of the Ca^2+^ Concentration

**Figure 2:**
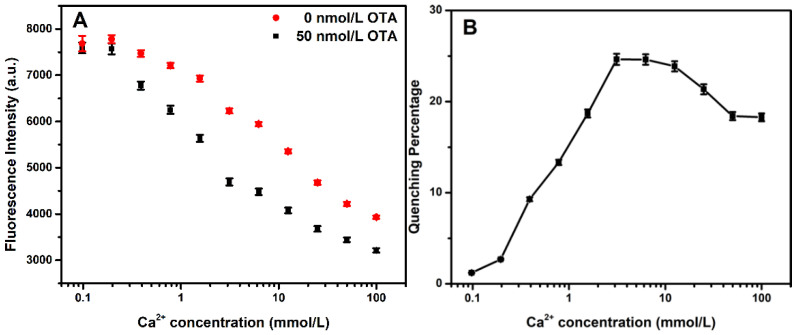
(**A**) Fluorescence intensity of the F1 aptasensor with different concentrations of Ca^2+^ (0–100 mmol/L) in the presence and absence of 50 nmol/L OTA (*n* = 3). (**B**) Quenching percentage with different concentrations of Ca^2+^ (0–100 mmol/L). The error bars denote the standard deviations of the triplicate measurements.

The important parameters were first optimized to obtain the optimal performances of the two proposed sensors. It is worth noting that the composition and pH of the buffer directly affect the aptamer structure in the solution. The Tris-HCl buffer is commonly used in biological experiments because nucleic acids are more stable in this solution, and the reaction is mild. Hence, Tris-HCl pH 8.4 was chosen to perform the following experiments. In addition, studies have shown that bivalent ions can shield the negative charge on the phosphate skeleton of nucleic acids, contributing to the formation of the three-dimensional recognition structure of the aptamer [19]. The effect of the Ca^2+^ ion concentration on the change in fluorescence intensity in the presence and absence of OTA was studied. By increasing the Ca^2+^ concentration, a rapid increase in the fluorescence quenching percentage ((F_0_-F_OTA_)/F_0_ ∗ 100) was observed. The fluorescence quenching reached a maximum at a concentration of 3.0 mmol/L. Therefore, the 3.0 mmol/L Ca^2+^ ion concentrations were chosen for the subsequent experiments, and the results are shown in Figure 2.

#### 3.2.2. Optimization of the Complementary Sequence and Its Concentration for F2 Aptasensor

**Figure 3:**
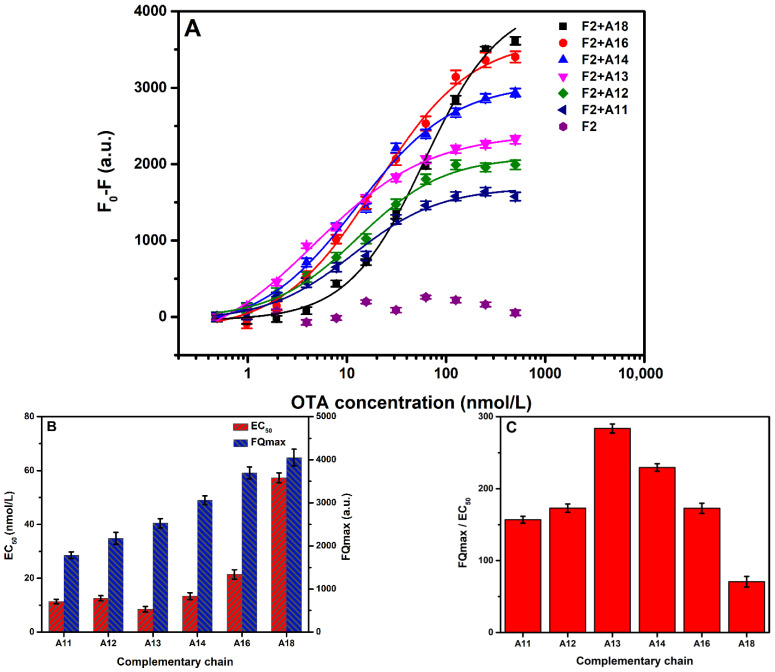

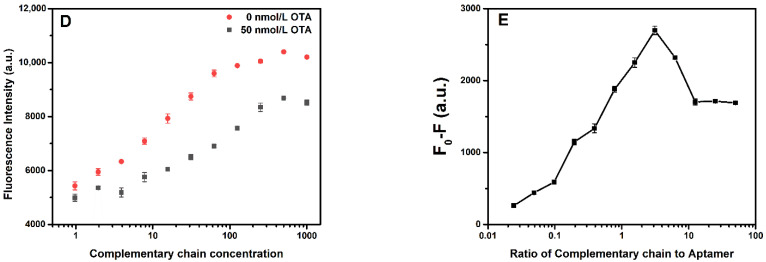
(**A**) Fluorescence quenching (F_0_-F) with different concentrations of OTA. (**B**) EC50 of the sensor and the maximum values of fluorescence quenching (FQmax) using different complementary sequences. (**C**) Sensitivity evaluation of the sensor using different complementary sequences. (**D**) Fluorescence intensity with different concentrations of the complementary chain (A13) (0–1000 nmol/L) in the absence and presence of 50 nmol/L OTA. (**E**) Fluorescence quenching (F_0_-F) with the ratio of the complementary chain (A13) to aptamer The error bars denote the standard deviation of the triplicate measurements.

The newly designed F2-based aptasensor shows no significant fluorescence intensity change when adding different concentrations of OTA. In order to solve this problem, complementary chains were introduced into the system to enhance the fluorescence intensity of F2. In a competition reaction, there is no doubt that the binding of a competitor to the aptamer will reduce the affinity of the aptamer for the target, thus increasing the detection limit of the sensor. Therefore, seven complementary chains were designed to obtain the best performance of the sensor. The number of bases in the complementary chain influenced the detection performance of the sensor. The calibration curves of seven complementary chains are shown in Figure 3A. The maximum values of the fluorescence quenching (FQmax) and EC50 of the aptasensors were obtained by fitting the calibration curves with a four-parameter equation according to origin, as follows

(1) $$\mathrm{FQ}=\frac{\mathrm{FQ}\min-\mathrm{FQ}\max}{1+(C_{\mathrm{OTA}}{/\mathrm{EC}50)}^{p}}+\mathrm{FQ}\max$$

As shown in Figure 3B, compared with the FQmax for different complementary chains, the increase in the number of the bases enhanced the fluorescence quenching. The EC50 value represented the strength of the interaction between the aptamer and the target molecule inhibited by different complementary strands. Hence, the number of bases in the chain promoted competitiveness, inhibited OTA binding to aptamers, and decreased the binding constant. Thus, to select the best complementary strand, (FQmax/EC50) was used to evaluate the sensitivity of the aptasensor, as shown in Figure 3C. The evaluation value of A13 was significant. Therefore, the sensor constructed with A13 provided a good aptasensing performance.

The complementary chain with an F2-based aptasensor in the system was in a competitive relationship with OTA, and its concentration directly affected the competitive ability and detection effect. Therefore, the optimal concentration of the complementary strand (A13) was determined by measuring the difference in fluorescence intensity in the presence and absence of OTA. The experiment investigated the fluorescence intensities of F2 with different concentrations of A13 in 50 nmol/L and 0 nmol/L of OTA. It was observed that the fluorescence intensity increased with the increase in concentration of A13, as shown in Figure 3D. As for A13, within a specific concentration range, the difference in fluorescence intensity between 50 nmol/L and 0 nmol/L of OTA increased with the increase in the concentration ratio. Accordingly, the fluorescence quenching (F_0_-F) also increased with the rise in the molar concentration ratio of A13/F2. When the molar concentration ratio was about 3, the fluorescence quenching decreased with the molar concentration ratio. It is worth noting that the higher concentration of the complementary strand enhanced hybridization with the aptamer and reduced the fluorescence difference. Therefore, a complementary chain with a molar concentration ratio of about 3 (60 nmol/L) was selected. The results are shown in Figure 3E.

### 3.3. Quantitative Analysis of OTA

**Figure 4:**
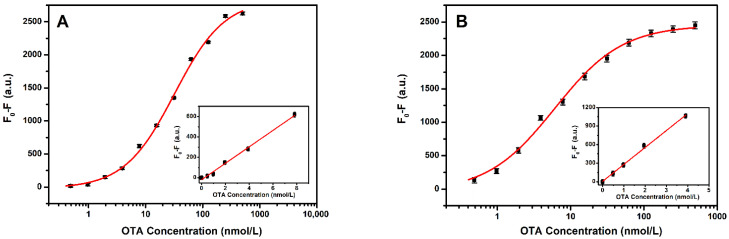
(**A**) F1 calibration curve for OTA (*n* = 3). (**B**) F2 with complementary chain (A13) calibration curve for OTA (*n* = 3). The inset shows the linearity of the fluorescence quenching (F_0_-F) with respect to the OTA concentrations. The F1 and F2 aptamer, and complementary chain concentrations were 20 nmol/L and 60 nmol/L, respectively. The error bars represent the standard deviation of the triplicate measurements.

Under the optimized conditions, the sensitivity of the proposed aptasensors was investigated. The sensing performance of the sensor for signal amplification was assessed by adding different concentrations of OTA. Figure 4A shows the calibration curve for the quantitative measurement of OTA using an F1-based aptasensor. As shown in the figure, a linear relationship between the fluorescence quenching (F_0_-F) and OTA concentration was obtained in the range of 0.69 to 8.0 nmol/L. The linear regression equation corresponded to fluorescence quenching (F_0_-F) = 80.9 C_OTA_ (nmol/L) − 14.4, with a correlation coefficient of 0.982. The limit of detection (LOD) was calculated as 0.69 nmol/L, as per the 3σ rule. Figure 4B shows the calibration curve for the quantitative detection of OTA using F2 with an A13 aptasensor. A linear relationship between the fluorescence and OTA concentration was observed, ranging from 0.36 to 4.0 nmol/L. The linear regression equation corresponds to the fluorescence quenching (F_0_-F) = 274.8 C_OTA_ (nmol/L) + 6.5, with a correlation coefficient of 0.998. The limit of detection (LOD) was calculated to be 0.36 nmol/L, as per the 3σ rule. Compared with the performance of the developed detection platforms, the sensitivity of F2 with the A13 aptasensor was 3.4 times that of F1. However, the F1 aptasensor was simpler to operate.

The sensing platform developed here was compared to other previously reported detection approaches for ochratoxin A (Table 2). As indicated in Table 2, the method developed here exhibited a lower LOD of 0.36 nmol/L than the techniques using high-affinity competitive substances such as gold nanoparticles (11.6 nmol/L) [26] and SWNTs (24.1 nmol/L) [22]. Indeed, the sensitivity of our biosensor was higher than that of the methods requiring enzyme amplification (10 nmol/L) [4,27] and that of the quencher-free fluorescence sensors (1.3 nmol/L) [13].

### 3.4. Specificity Analysis and Application to Real Samples

**Figure 5:**
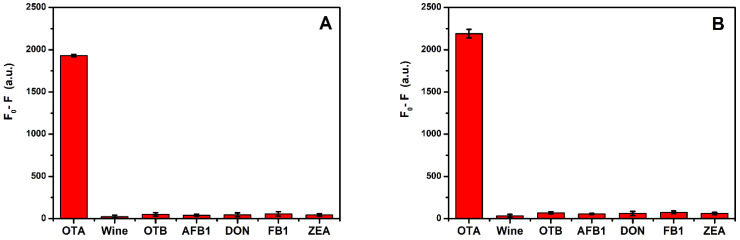
(**A**) Selectivity of the F1 aptasensor (*n* = 3). (**B**) Selectivity of the F2 with A13 aptasensor (*n* = 3). Fluorescence quenching (F_0_-F) of 62.5 nmol/L OTA and 250 nmol/L OTB, AFB1, DON, FB1, and ZEA. The error bars denote the standard deviation of the triplicate measurements.

The specificity of the constructed aptasensor for OTA was tested in a real sample. In addition, the system was applied to detect other common analogs, including OTB, AFB1, DON, FB1, and ZEA. The relevant detection process has been described in various reports [25]. The detection results are shown in Figure 5. The fluorescence quenching of 250 nmol/L of interference was much lower than for 62.5 nmol/L of OTA. These results demonstrate the excellent specificity and selectivity of the proposed platform for OTA determination.

The applicability of this sensing system for real sample analysis for OTA determination was conducted by standard addition and recovery analysis in actual red wine samples. The relevant detection process has been described in various reports [25]. The pre-treatment of the sample was performed using the scheme of double-liquid–liquid extraction, which was explicitly designed according to the chemical properties of OTA. OTA is only soluble in the aqueous phase under alkaline conditions, while it is soluble in the organic phase under acidic conditions. Therefore, cross-reactivity with interferents and other matrix components could be overcome in pre-treated red wine samples, even for some molecules with similar chemical properties to OTA. Therefore, the effective and specific detection of OTA in wine can be achieved by combining sample pre-treatment and fluorescent aptasensing.

To show the potential applicability and accuracy of the sensing strategy, the recovery rates were calculated based on three-level concentrations of OTA spiked in red wine samples. Excellent recoveries were obtained, varying between 88.7% and 107.7% by using the F1 aptasensor and between 95.7% and 104.3% using the F2 with A13 aptasensor in the samples spiked with OTA. These results demonstrate that the proposed method could be applied to OTA detection in real complex samples. The corresponding results are displayed in Table 3.

This kit can meet the maximum permissible concentration at 2 μg/kg (corresponding to 5 nmol/L), as specified in European Commission No. 123/2005 [20]. It also provides promising potential for portable detection and in-field monitoring of small molecules such as OTA.

## 4. Conclusions

To summarize, a rapid and straightforward sensing platform is developed for the sensitive and selective detection of OTA based on a quencher-free fluorescence approach by a FAM-labelled aptamer probe. The detection principle relies on the high sensitivity, specificity, and high affinity between OTA and its aptamer, as well as the quenching ability of guanine induced by PIET. By comparing the performances of the two aptasensors, the sensitivity of F2 with the A13 aptasensor (LOD is 0.36 nmol/L) was better than that of the F1 aptasensor (LOD is 0.69 nmol/L), while the F1 aptasensor was simpler to perform. The kits developed here based on the two designed aptasensors can meet the maximum permissible concentration at 2 μg/kg (corresponding to 5 nmol/L), as specified in European Commission No. 123/2005 [20]. The proposed strategy provides fast response, low cost, and high sensitivity and selectivity for OTA detection. It is simple and straightforward. It also shows good potential to be integrated into portable systems, which will facilitate on-site mycotoxin screening. In addition, the strategy of using a single-labeled fluorophore and guanine as a quencher without the necessity for a labeled quencher may be applied to detect various other targets in different fields, such as in environmental monitoring and food safety fields.

## Figures and Tables

**Table 1:** DNA sequences used in this experiment.

Name	Complementary Chain Sequence	Number of Bases
Apt36F	5′-GGGGATCGGGTGTGGGTGGCGTAAAGGGAGCATC-GGACA-FAM-3′	39
F1	5′-GGGGATCGGGTGTGGGTGGCGTAAAGGGAGCATC-FAM-3′	34
F2	5′-GGGGGATCGGGTGTGGGTGGCGTAAAGGGAGCATCC-FAM-3′	36
F2-A11	3′-TTTCCCTCGTA-5′	11
F2-A12	3′-ATTTCCCTCGTA-5′	12
F2-A13	3′-CATTTCCCTCGTA-5′	13
F2-A14	3′-GCATTTCCCTCGTA-5′	14
F2-A16	3′-CCGCATTTCCCTCGTA-5′	16
F2-A18	3′-CACCGCATTTCCCTCGTA-5′	18

**Table 2:** Comparison of the analytical performance of the analytical methods for the detection of OTA.

Method/Material	Detection Limit (nmol/L)	Linear Range (nmol/L)	Reference
Fluorescence SYBR gold probe and exonuclease III	11.6	19.8–2476	[4]
Fluorescence PIET system	1.3	3–300	[13]
Fluorescence single-walled carbon-nanotubes (SWCNTs)	24.1	25–200	[22]
Fluorescence gold nanoparticles	22.7	25–300	[26]
Colorimetry G-quadruplex DNAzymes	10	10–200	[27]
Electrochemistry polythiophene-3-carboxylic acid	0.31	0.31–12.4	[28]
Quencher-free fluorescence (F1)	0.69	0.69 to 8.0	This work
Quencher-free fluorescence (F2 with A13)	0.36	0.36 to 4.0	This work

**Table 3:** Detection of OTA in red wine (*n* = 3).

Method	Sample	Found (nmol/L)	Added (nmol/L)	Total Found (nmol/L)	Recovery (%)
F1	1	Not detected	4.0	3.89 ± 0.28	97.3
F1	2	Not detected	2.0	2.15 ± 0.26	107.7
F1	3	Not detected	1.0	0.89 ± 0.13	88.7
F2 + A13	4	Not detected	4.0	3.85 ± 0.12	96.2
F2 + A13	5	Not detected	2.0	2.09 ± 0.15	104.3
F2 + A13	6	Not detected	1.0	0.96 ± 0.12	95.7

## Data Availability

Not applicable.
